# Review of Asherman syndrome and its hysteroscopic treatment outcomes: experience in a low-resource setting

**DOI:** 10.1186/s12905-024-02944-0

**Published:** 2024-02-07

**Authors:** Melkamu Siferih, Thomas Gebre, Fufa Hunduma, Abraham Abebe, Akebom Gebremichael, Habtamu Sewunet, Tewodros Shibabaw

**Affiliations:** 1 Department of Obstetrics and Gynecology, School of Medicine, Debremarkos University, Debremarkos, Ethiopia; 2 Reproductive Endocrinology and Infertility, Department of Obstetrics and Gynecology, St. Paul’s Hospital Millennium Medical College, Addis Ababa, Ethiopia; 3 Field Epidemiology, Department of Public Health, St. Paul’s Hospital Millennium Medical College, Addis Ababa, Ethiopia; 4 Department of Obstetrics and Gynecology, St. Paul’s Hospital Millennium Medical College, Addis Ababa, Ethiopia; 5 Department of Midwifery, Debremarkos Comprehensive Specialized Hospital, Debremarkos, Ethiopia; 6 School of Medicine, College of Medicine and Health Sciences, Gondar University, Gondar, Ethiopia

**Keywords:** Asherman syndrome, Hysteroscopic adhesiolysis, Treatment outcomes, Ethiopia

## Abstract

**Background:**

Asherman syndrome is one of the endometrial factors that influence a woman’s reproductive capacity. However, in our context, it needs to be well-documented. This study aimed to evaluate the clinical characteristics and hysteroscopic treatment outcomes of Asherman syndrome.

**Method:**

A retrospective follow-up study from January 1, 2019, to December 31, 2022, was conducted on cases of Asherman syndrome after hysteroscopic adhesiolysis at St.Paul’s Hospital in Addis Ababa, Ethiopia. Clinical data were collected via telephone survey and checklist. Epidata-4.2 and SPSS-26 were employed for data entry and analysis, respectively.

**Result:**

A total of 177 study participants were included in the final analysis. The mean patient age was 31 years (range: 21–39) at the initial presentation, and 32.3 years (range: 22–40) during the phone interview. The majority of the patients (97.7%) had infertility, followed by menstrual abnormalities (73.5%). Among them, nearly half (47.5%) had severe, 38.4% had moderate, and 14.1% had mild Asherman syndrome. The review identified no factor for 51.4% of the participants. Endometrial tuberculosis affected 42 patients (23.7%). It was also the most frequent factor in both moderate and severe cases of Asherman syndrome. Only 14.7% of patients reported menstrual correction. Overall, 11% of women conceived. Nine patients miscarried, three delivered viable babies, and six were still pregnant. The overall rate of adhesion reformation was 36.2%. Four individuals had complications (3 uterine perforations and one fluid overload) making a complication rate of 2.3%.

**Conclusion:**

Our study revealed that severe forms of Asherman syndrome, which are marked by amenorrhea and infertility, were more common, leading to incredibly low rates of conception and the resumption of regular menstruation, as well as high recurrence rates. A high index of suspicion for Asherman syndrome, quick and sensitive diagnostic testing, and the development of a special algorithm to identify endometrial tuberculosis are therefore essential. Future multi-centered studies should focus on adhesion preventive techniques.

## Background

In 1948, Joseph Asherman coined the term “Asherman syndrome(AS),” which is an acquired endometrial condition defined by avascular myofibrous intrauterine adhesions (IUAs), and at least one of the following symptoms: history of reduced fertility, repeated miscarriages, dysmenorrhea, non-cyclic pelvic pain, aberrant placentation, or menstrual irregularities (amenorrhea, hypomenorrhea, or oligomenorrhea) [1–3].

Uncertainty surrounds IUA’s precise pathophysiological genesis. However, pregnancy is the factor that is most usually mentioned as having occurred before Asherman syndrome (AS). It occurs after the endometrial layer, particularly the basal layer of the endometrium, has been damaged, causing the two opposing surfaces of the uterus to fuse. The adhesions may involve the endometrium, myometrium, or multiple layers of connective tissue [2, 4, 5]. In the AS case analysis, abortion/miscarriage curettage (66.7%) and postpartum curettage (21.5%) were the two most significant factors [6]. There have also been other etiologic factors reported, such as genital tuberculosis (TB), intrauterine devices, myomectomy, uterine surgery such as cesarean sections, diagnostic curettage, and hysteroscopic surgery [2, 4, 5].

Although it is impossible to determine the exact prevalence of IUA, it is uncommon and frequently asymptomatic in the general population [7, 8]. It varied between 1.5% in cases of infertility and 30% following intrauterine instrumentation [1, 9–11]. However, in patients with a history of endometrial tuberculosis (TB), the incidence rate reached 67.8% [11, 12]. Menstrual abnormalities, mainly hypomenorrhoea or secondary amenorrhea, are common symptoms of AS. In cases where other people have pretty normal periods, a high level of suspicion is required to make a diagnosis [7, 13].

Before the advent of hysteroscopy, hysterosalpingography, and saline infusion sonohysterography were considered the diagnostic methods of choice for visualization of the uterine cavity [14, 15]. Hysteroscopy can more accurately confirm the presence, extent, and degree of adhesions and the quality of the endometrium. Hysteroscopic adhesiolysis (HA) is currently the treatment of choice for AS because of its minimally invasive nature and because it can be performed under image guidance [16]. In most cases, adhesiolysis can be done by hysteroscopic scissors or other cutting methods such as laser or diathermy. Adhesiolysis scissors or biopsy forceps have the advantage of being able to make an incision, avoiding complications in energy sources, and minimizing endometrial destruction [14, 17].

On the other hand, two clinical practice tasks are endometrial repair and preventing recurrent IUA. The two most crucial elements in preventing the recurrence of adhesions are endometrial growth-promoting estrogens and physical barriers that support uterine shape [18]. Recently, IUD insertion, high-dose estrogen therapy, and antibiotics have been used in combination to prevent recurrent adhesions and promote endometrial repair and regeneration. Most IUDs are placed in the uterine cavity for 2–3 months [7, 8, 19, 20]. The recovery of normal uterine cavity anatomy, adhesion reformation, restoration of regular menstruation, pregnancy rate, and live birth rate are used to evaluate the success of HA [21, 22].

To the best of our knowledge, Ethiopia has no data on Asherman syndrome. The country has extremely few resources for hysteroscopic adhesiolysis, including data, availability, and practice. The only public Center for Fertility and Reproductive Medicine (CFRM) in our system at St. Paul’s Hospital currently performs hysteroscopic adhesiolysis.

To identify patients during postoperative follow-up and prognosis counseling, physicians will need to review AS factors, clinical characteristics, and treatment outcomes.

The current study aimed to assess the clinical characteristics of Asherman syndrome and its hysteroscopic treatment outcomes at CFRM in St. Paul’s Hospital.

## Methods and materials

### Study design, period, and participants

A retrospective follow-up study was carried out at the Center for Fertility and Reproductive Medicine in St. Paul’s Hospital in Addis Ababa, the capital city of Ethiopia, between January 1, 2019 and December 31, 2022. The CFRM is the only public center in the country that provides ART services to infertile couples and performs laparoscopy, hysteroscopy, and other minimally invasive procedures. Since its establishment (January 1, 2019), more than 68,000 couples have been observed in the fertility center. The center has six infertility subspecialists. All HA was carried out by subspecialists in infertility with at least five years of expertise. Hysteroscopy was done in the operating room. Patients were mainly under general anesthesia. For operative hysteroscopy, dilatation of the cervix was required to insert an 8- to 10-mm hysteroscope to ease dilatation and lower the risk of uterine perforation; cervical preparation was made by using misoprostol. Both diagnostic and therapeutic hysteroscopies were performed with a hysteroscope consisting of a 4 mm outer sheath and a rigid telescope Bettocchi (2.9 mm, up to 30° continuous flow; Karl Storz, Tuttlingen, German). Hysteroscopic scissors were used for adhesiolysis. The procedure was performed under the guidance of transabdominal ultrasound. The distension medium was normal saline or Ringer’s lactate instilled at a controlled pressure of 70 to 80 mm Hg and rarely more than 100 mmHg for both diagnostic and operative hysteroscopy. All the procedures were monitored with a video camera and monitor. Each patient underwent a hysteroscopic-directed biopsy to ensure that no endometrial abnormalities were missed. A pale area on the uterine wall and caseated uterine cavity during hysteroscopy could raise suspicions that tuberculosis-related adhesions were to blame. Finally, it was verified through endometrial biopsy (histology or culture).

A pediatric Foley catheter was inserted for 7–10 days after surgery, and estrogen with progesterone or estradiol valerate (4–8 mg/day) was given for 4 weeks. For the final seven days of the estrogen therapy, medroxyprogesterone acetate (10 mg) was given orally daily. For two to three months, copper IUDs were placed. Control hysteroscopy was made in the early proliferative phase, 1–2 months after the first surgery. The degree and severity of adhesions were assessed during the reexamination, and HA was done if adhesions reappeared.

The source population was patients who underwent hysteroscopic adhesiolysis at the Center for Fertility and Reproductive Medicine. The study population consisted of patients who met the criteria for inclusion and underwent HA during the study period. Patients with HA at least two months before, those between the ages of 20 and 40 at the time of the initial presentation and data collection, those who were successfully contacted and completed the telephone survey, and provided verbal informed consent were all included. Women who had not had a follow-up hysteroscopy two months after HA, other proven causes of infertility, or intrauterine conditions (such as endometrial hyperplasia, polyps, adenomyosis, or uterine abnormalities) were excluded. Patients were selected from the institution’s medical records and asked to complete a telephone interview script. Consecutive sampling was used to choose study participants overall.

Using criteria such as the degree of uterine cavity involvement, the characteristics of intrauterine adhesions, and the characteristics of the menstrual cycle, the stage of AS was described with an appropriate score following the principles for the Asherman syndrome system of the American Society of Reproductive Medicine (ASRM) [23]: AS ranges from mild (score 1–4), moderate (score 5–8), to severe (score 9–12).

### Study variables

**The outcome variables** were the resumption of menstruation, the occurrence of conception, abortion, live births, cessation of pelvic pain, recurrence of adhesion, intraoperative complications, and stages of disease severity after treatment. **Independent variables** included sociodemographic variables such as ages (in years) at presentation and during data collection, address, parity, marital status, educational attainment, religion, and occupation. Moreover, clinical characteristics such as possible factors, infertility, menstrual abnormalities (amenorrhoea, hypomenorrhoea, dysmenorrhea, and oligomenorrhea), non-cyclic pelvic pain, recurrent pregnancy loss, stages of disease severity before treatment (mild, moderate, and severe Asherman syndrome), and use of antibiotics were included.

### Data collection tool and data quality assurance

A structured English version data abstraction checklist and scripted telephone survey were developed by using different published articles [1, 2, 4, 5, 7–11, 24–26]. The English version scripted telephone survey was translated into the local language, the Amharic version, which was the language spoken by the study participants. It was then back-translated into an English version by another expert to check for consistency. It was filled by trained data collectors. Two BSC nurses and one medical doctor were recruited for data collection, supervision, and patient classification based on disease severity, respectively. Data collectors were given training by the principal investigator about the goal of the research and data collection procedures. The disease severity of the patients was classified via the American Society of Reproductive Medicine system. After taking their mobile number from their charts, patients were contacted and asked to fill out the telephone survey script. The checklist and the scripted telephone survey were pretested on 10% of patients one week before the main data collection. Patients in the same area were included in the pretest. The checklist and the telephone survey script were modified for clarity, sensitivity, and completeness based on the pretest data. The quality of data was assessed every day after data collection.

### Operational definition

The patient’s current age was the age at which the data was collected. Early and late second-look hysteroscopy refers to hysteroscopic examinations performed within the first two months after adhesiolysis and two months afterward, respectively. A period lasting less than two days or spot bleeding was a symptom of hypomenorrhea. Abortion or miscarriage has been defined as the loss of pregnancy before 28 weeks of gestation. Oligomenorrhea occurred more than 35 days apart. Two or more consecutive miscarriages before 28 weeks of pregnancy were considered recurrent pregnancy loss.

### Data processing and statistical analysis

Data were double entered and cleaned, with Epidata version 4.2 statistical and exported to the SPSS-26 version for analysis. The data were collected, summarized, tabulated, analyzed, and expressed with descriptive statistics such as frequency, percentage, mean, median, and interquartile range. For categorical variables, frequencies and percentages were reported; for continuous variables with normally distributed data, mean; and for data with a non-normal distribution, median and interquartile range. The Chi-square test and Fisher’s exact test were used to show associations between treatment outcomes and disease severity. Posthoc tests with Bonferroni residual analysis were done after a significant overall chi-squared test to determine where the difference is. Overall *p* value < 0.05 and Bonferroni adjusted *p* values were considered for statistical significance.

### Result

#### Sociodemographic and clinical characteristics

Out of the 1338 gynecologic procedures performed in the center during the study period, 233 patients (17.4%) had hysteroscopic adhesiolysis for Asherman syndrome. Of these, 202 (86.7%) patients met the inclusion criteria. However, 177(87.6%) patients were included in the final analysis. The mean patient age was 31 years (range: 21–39 years) at the time of their presentation, and 32.3 years (range: 22–40) during their phone interview. Of the study participants, 98.3% were married and 93.2% resided in urban areas. Besides, the majority (70.6%) of study participants were parous, and 44.6% had at least a secondary level of education (Table 1).

**Table 1:** Sociodemographic characteristics of patients who underwent hysteroscopic adhesiolysis

Patient characteristics	Category	(*N* = 177) (n, %)
Address	rural	12(6.8)
	urban	165(93.2)
Religion	Orthodox	101(57.1)
	Muslim	55(31.1)
	Protestant	21(11.9)
Occupation	housewife	54(30.5)
	civil servant	65(36.7)
	merchant	58(32.8)
Marital status	married	174(98.3)
	single	3(1.7)
Educational status	no formal education	5(2.8)
	primary School	25(14.1)
	secondary School	79(44.6)
	tertiary level	68(38.4)
Parity	nulliparous	52(29.4)
	parous	125(70.6)

The study did not detect any factor for 51.4% of patients. Endometrial tuberculosis was diagnosed in 42(23.7%) cases of AS. It accounted for 48.8% of all known possible factors (Table 2). Additionally, patients with mild (26.2%) and severe (71.4%) AS had it as their most common factor. Endometrial TB was detected by histology (tuberculous granuloma) in 91% of cases, by positive culture on endometrial biopsy in 2% of cases, and by hysteroscopy in 7% of patients (which revealed a caseated uterine cavity and pale patches).

**Table 2:** Possible factors

Factors	(*N* = 177) (n, %)
Unknown/no obvious factor	91(51.4)
Post abortal curettage	10(5.6)
Cesarean section	14(7.9)
Myomectomy	6(3.4)
Endometrial TB	42(23.7)
Recurrent pregnancy loss	14(7.9)

Infertility was identified in virtually all patients (97.7%) at presentation, followed by menstrual abnormalities (73.5%). The most frequently reported issue was primary infertility (56.5%), followed by secondary infertility (41.2%) and amenorrhea with infertility (41.2%). Amenorrhea was the most typical menstrual pattern (38.4%). Fourteen patients (7.9%) had recurrent pregnancy loss (Table 3).

**Table 3:** Initial patient complaints with Asherman syndrome

Presenting complaint	Category	(*N* = 177) (n, %)
Infertility	primary infertility	100(56.5)
	secondary infertility	73(41.2)
Recurrent pregnancy loss	yes	14(7.9)
	no	163 (92.1)
Menstrual abnormality	amenorrhoea	68(38.4)
	oligomenorrhea	21(11.9)
	hypomenorrhoea	41(23.2)
Normal menses	yes	46(26.0)
	no	131 (74)
Pelvic pain	dysmenorrhea	12(6.8)
	non-cyclic pelvic pain	31(17.5)
Oligomenorrhea and infertility both	yes	20(11.3)
	no	157 (88.7)
Hypomenorrhoea and infertility both	yes	36(20.3)
	no	141 (79.7)
Amenorrhoea and infertility both	yes	73(41.2)
	no	104 (58.8)

According to the ASRM classification of AS with its severity, our study clearly showed that nearly half (47.5%) of patients had severe disease, followed by 38.4% who had moderate disease, and 14.1% who had mild disease. The range of complaint duration was 3-240 months, with a mean (SD) duration of 71.7(49.9) months.

The majority of patients (97.7%) were prescribed prophylactic antibiotics, such as ampicillin, ceftriaxone, or doxycycline, but for the rest of the patients, there was no documentation about antibiotics. Additionally, they were given estradiol valerate (96%), copper IUCD (92.7%), oral medroxyprogesterone acetate (83.6%), ant-TB (23.7%), COC (3.4%), and vaginal sildenafil citrate (3.4%). One-to three-month adjuvant therapy was administered to 76.3% of patients, with six-month adjuvant treatments being administered to the remainder of individuals. Their mean (SD) follow-up time in months was 7.2(9.3) (range: 2–72).

## Reproductive/obstetric outcomes

One-hundred seventy-three patients needed assisted reproductive technology; of them, 48.6% had severe AS, 38.7% had moderate AS, and 12.7% had mild AS. Nineteen of them reported an overall conception rate of 11% (overall *p*.value = 0.015). The observed difference in conception rate truly came from stage III (severe) Asherman syndrome (adjusted *p*-value = 0.0013) (Table 4).

**Table 4:** Preoperative Asherman syndrome severity and conception rate (adjusted Bonferroni *p*-value = 0.008)

Preoperative Disease Severity	Conception (n, %)		(*N* = 173)	Overall *p*-value
	yes	no	total	
Stage I(mild)	6(27.3)	16(72.7)	22	0.015
Stage II(moderate)	9(13.4)	58(86.6)	67	
Stage III(severe)	4(4.8)	80(95.2)	84	

Nine patients required IVF, and just ten naturally conceived. The median time to conception was six months (IQR: three to fifteen months). At the time of the telephone interview, six patients (31.6%) had ongoing pregnancies, of which two were in the third trimester, one was in the second, and three were in the first. Nine patients (47.4%) had miscarriages, and three (15.8%) had live births.

Hypomenorrhea and amenorrhea at initial presentation had low conception rates (4.9% and 7.4%, respectively). There was a significant association between presenting menstrual patterns and conception rate. The paired comparison of hypomenorrhea and conception rate was the primary source of the difference (adjusted *p*-value = 0.003) (Table 5).

**Table 5:** Pregnancy rate determined by presenting menstrual pattern (adjusted Bonferroni *p*-value = 0.0063)

Presenting menstrual pattern	Conception (n, %)		(*N* = 173)	Overall *p*-value
	yes	no	total	
Normal menses	10(23.3)	33(76.7)	43	0.04
Hypomenorrhoea	2(4.9)	39(95.1)	41	
Oligomenorrhea	2(9.5)	19(90.5)	21	
Amenorrhoea	5(7.4)	63(92.6)	68	

### Menstrual outcomes

Overall, 14.7% of patients had menstrual correction. Whereas 27.7% of women suffered amenorrhea, 15.8% of women maintained their regular menstrual cycles (Table 6).

**Table 6:** Menstrual outcomes following Asherman syndrome treatment

Menstrual Pattern after Adhesiolysis	(*N* = 177) (n, %)
No change in previous normal menses Achieved normal menses Slight improvement in normal menses	28 (15.8)
	26(14.7)
	7(3.9)
Hypomenorrhoea	41(23.2)
Oligomenorrhea	26(14.7)
Amenorrhoea	49(27.7)

After hysteroscopic adhesiolysis, 50% of the 68 women who initially presented with amenorrhea still had it; 16.2% of them had normal menstruation, 1.5% had just slightly improved 17.6% had hypomenorrhoea and 14.7% had oligomenorrhea. Among patients with severe disease at the time of presentation, 20.2% had hypomenorrhea, 16.7% had oligomenorrhea, 13.1% had normal menses, 3.6% exhibited some improvement, and 46.4% were amenorrheic after treatment.

### Recurrences and complications

91% of patients had an early second-look hysteroscopy, and the rest had a late second-look after hysteroscopic adhesiolysis.

A considerable amount of adhesion was present in 36.2% of the women during the second-look hysteroscopy, with incidences in mild, moderate, and severe AS were 9.1%, 18.6%, and 8.5%, respectively. Women with mild, moderate, and severe preoperative AS experienced recurrence rates of 18.5%, 24.6%, and 50.6%, respectively. Due to the stage II disease and the existence of significant adhesion, there was a significant association between the preoperative disease severity and adhesion rate ((adjusted *p*-value = 0.003) (Table 7).

**Table 7:** Second-look hysteroscopy findings and preoperative disease severity (adjusted Bonferroni *p*-value = 0.008)

Preoperative Disease Severity	Significant adhesion (n, %)		*N* = 177	Overall *p*.value
	no	yes	total	
**Stage I disease**	22(81.5)	5(18.5)	27	0.001
Stage II disease	49(75.4)	16(24.6)	65	
Stage III disease	42(49.4)	43(50.6)	85	

Recurrence rates were 4.5%, 8.5%, and 23.2% for uterine cavity involvement of one-third, between one-third and two-thirds, and more than two-thirds, respectively. When the intrauterine adhesion was filmy, hard, and merely dense, the rates of recurrence were 2.3%, 4%, and 29.9%, respectively. Hysteroscopic adhesiolysis was performed once for 9.6%, twice for 22%, and three times for 3.4% of the patients with recurrent intrauterine adhesions.

Complications occurred in 4 patients (2.3%). In three of these patients, uterine perforation took place during adhesion dissection for severe AS, while in the fourth patient, fluid overload complicated mild AS. Two uterine perforations occurred during repeated adhesiolysis. In this review, there were no reports of complications such as false passage, cervical tear, endometritis, or embolism. After adhesiolysis, 15.8% of patients had pelvic pain, of which 9% reported non-cyclic pelvic pain and 6.8% reported dysmenorrhea.

## Discussion

Asherman syndrome is a well-documented gynecologic condition associated with a high rate of infertility, menstrual irregularity, miscarriage, or abnormal placentation. In this research, we studied the clinical characteristics and treatment outcomes of Asherman syndrome as well as intraoperative complications and recurrence after hysteroscopic adhesiolysis at St. Paul’s Hospital Millennium Medical College Center for Fertility and Reproductive Medicine.

Despite the difficulty in pinpointing the exact prevalence of Asherman syndrome, the 17.4% prevalence in our study was in the range of 1.5% in instances of infertility to 30% after intrauterine instrumentation [1, 9–11]. The majority of patients’ factors (51.4%) could not be investigated. This can be due to a scarcity of in-depth investigations. This study showed that endometrial tuberculosis was the most prevalent factor, accounting for 48.8% of all known possible etiologies. Pregnancy-related factors, such as cesarean sections, postabortal curettage, and recurrent pregnancy loss, were in second place (44.2%). While pregnancy-related factors and endometrial TB both contributed to Asherman syndrome in our study, endometrial TB alone accounted for 23.7% of cases. Prior research [2, 4–7] found that the most common factor was pregnancy-related, such as postabortal curettage, but our conclusion contrasts with those findings. This disparity implied that developing countries like Ethiopia had high tuberculosis prevalence [27–29]. This calls for the creation of a unique algorithm to detect genital TB by a national team. In such nations lacking resources for mycobacterial culture and histology, diagnosis is frequently restricted to clinical suspicion. The lack of conclusive histology makes diagnosis challenging even in situations where these resources are accessible [30]. Our findings, however, are in line with those of other studies conducted in Nigeria, India, and Pakistan [11, 12, 27].

The most frequent clinical presentations in this study were infertility and irregular menstruation, which was in agreement with many other studies [5, 31–35]. The representative presentation was that of primary infertility. Compared to earlier findings, this one was significantly more important [32, 34, 35]. This may be because patients frequently came to our institution due to infertility. Patients with infertility difficulties are typically referred by other physicians from different areas of our country given that this is the center’s primary focus. Even though the review excluded demonstrable causes of infertility, the study might include study participants with unexplained causes of infertility. The other explanation may be there is a high burden of infectious diseases such as genital tuberculosis in the study area, which made primary infertility more dominant. Patients with menstrual irregularities usually presented with amenorrhea and hypomenorrhoea, which was in harmony with the preceding findings [5, 31, 33, 35, 36]. Taking this, many patients also had amenorrhea with infertility, followed by hypomenorrhoea with infertility, in addition to infertility and irregular menstruation alone. The outcome was distinct from what was discovered in Nigeria [7]. The inclusion of endometrial tuberculosis might help to explain the disparity. Amenorrhea and hypomenorrhea, in contrast, were more prevalent in severe illness than in mild to moderate illness. This finding is parallel with studies done in China, Malaysia, and Denmark [8, 16, 32, 37, 38].

Unlike previous studies [11, 26, 32, 34, 37, 39], our research findings showed that the pregnancy and live birth rates were lower for all Asherman syndrome individuals who tried to conceive after HA. This likely pertains to a shorter follow-up time in our study. The majority of patients had a severe disease classification at presentation, which will also have a marked statistical impact on post-HA outcomes. Moreover, the presence of endometrial tuberculosis-related intrauterine adhesions in the majority of our cases could also be considered a contributing factor. The miscarriage rate was generally higher for mild and moderate disorders; however, it was lower for severe diseases. This was not compatible with previously conducted research in Taiwanese women [32, 39]. This discrepancy between other studies might be explained by the extremely small percentage of patients in our setting with severe intrauterine adhesion who might be pregnant.

In addition, the likelihood of conception was declining as amenorrhea replaced normal menses. This is in keeping with the previous study that was published in China [37]. The normal residual endometrial function in people with reasonably normal menses may be the cause. This depicts that an important indicator of reproductive potential is endometrial function following HA.

In contrast to other research, the observed return to normal menstruation was lower, with the majority of cases ranging between 19.2% and 100% [24, 31, 40, 41]. The severe form of Asherman syndrome that our patients have may be the primary cause of the significant discrepancy between our results and the prior findings. The uterine cavities of the vast majority of our amenorrheic people may be almost nonexistent. In the circumstances we encountered, endometrial tuberculosis affected the endometrium’s inner layer and restricted its regeneration. In comparison to other research [24, 31, 34, 40, 41], only 16.2% of amenorrhoea resolved after HA. Since the majority of research did not make use of the ASRM classification method, it was thought that the disagreement may have resulted from the inclusion of menstrual patterns in the illness severity assessment. This is substantiated by the finding that patients with mild disease show a higher percentage of functional endometrium compared to those with other disease severity levels.

The intrauterine adhesion recurrence rate in our study was 36.2%, which was significant but still within the range of instances that had been previously recorded (27.3 − 69.6%,) [22, 24, 26]. Compared to the Indian report (6.9%) [42], this figure was higher. The inconsistency may be due to the absence of second-look protocols, the difference in adjuvant therapies concerning dosage and duration, the variation in the interval to the second-look hysteroscopy, the duration of the follow-up, and the predominance of infectious comorbidities among regional heterogeneity. Our patients had a higher rate of adhesion formation when they had a severe illness or high initial ASRM scores. This is in concordance with the previous studies [2, 24–26]. The rate of recurrence also rose in tandem with the preoperative degree of intrauterine involvement and the change from filmy to thick intrauterine adhesions. This finding agreed with the preceding reports [25, 43].

Expectant management with meticulous hemodynamic monitoring was employed in two uterine perforation cases. Exploratory laparotomy and anterior uterine repair were carried out in the third case. The patient with fluid overload initially appeared with non-cardiogenic pulmonary edema; subsequently, fluid restriction, and intravenous furosemide administration, a chest X-ray, and an echocardiogram were performed. Based on the existence of pulmonary edema symptoms, documented fluid deficit, and abnormalities on a chest X-ray, fluid overload was suspected. Each complication had similar symptoms, and we followed the same course of treatment as in the prior reports [44, 45]. Our research outcomes were comparable to those of several other reports [44, 46, 47], where hysteroscopic perforation was the most common complication, as well as Indian research with a 2.7% complication rate. However, it was higher than the prior studies, ranging from 0.8 to 1.6% [48–51]. This may be accounted for by the fact that the majority of our patients had severe intrauterine adhesions, which raised the possibility of perforation.

The utilization of both medical record review and telephone interviews is the strength of this study. The disease severity was rated using the ASRM categorization, a more objective classification that is frequently used and evaluates patient complaints by considering menstrual cycles. To the best of our knowledge, it is the first study that describes typical clinical characteristics, and treatment outcomes connected with Asherman syndrome in Ethiopia. The small sample size and single-centered focus are some of the study’s drawbacks. The outcomes of our study may not be generalizable due to its retrospective character. Recall bias will rise during patient reports via telephone interviews. The study was based on records or charts, some of which were lacking important data. There were no guidelines for adjuvant treatment dosage and duration, second-look hysteroscopy, or the length of patient follow-up. It was challenging to compare the findings of this study with those of the earlier studies since various disease classification systems and diagnostic and treatment approaches were employed in those earlier studies. Last but not least, the study could not review confounding factors such as technical differences, level of expertise, and effectiveness of each preventive method of Asherman syndrome.

### Conclusion and recommendation

Infertility and amenorrhea were the most frequent Asherman syndrome manifestations. Severe forms of Asherman syndrome that were more prevalent in our settings were the cause of a lower-than-expected rate of conception and restoration of regular menstruation, as well as a high rate of adhesion recurrences. Endometrial tuberculosis was the underlying factor in many cases of Asherman syndrome. Rapid and sensitive diagnostic tests and the development of a unique algorithm to detect endometrial tuberculosis, and a high index of suspicion for Asherman syndrome are crucial to reversing the disease states. Future multi-centered studies should focus on adhesion preventive techniques.

## Data Availability

Data will be available from the corresponding author upon reasonable request.

## Abbreviations

AS: Asherman Syndrome
ASRM: American Society of Reproductive Medicine
ART: Assisted Reproductive Technology
BSC: Bachelor of Science
CFRM: Center for Fertility and Reproductive Medicine
COC: Combined Oral Contraceptives
ERB: Ethical Review Board
HA: Hysteroscopic Adhesiolysis
IUA: Intrauterine Adhesion
IQR: Interquartile Range
IUCD: Intrauterine Contraceptive Devices
IVF: Invitro fertilization
MVA: Manual Vaccum Aspiration
SD: Standard Deviation
SPHMMC: Saint Paul’s Hospital Millennium Medical College
SPSS: Statistical Package for Social Sciences
TB: Tuberculosis
